# Intramuscular recurrence in a Hepatocellular carcinoma patient with indolent disease course

**DOI:** 10.1186/1477-7819-6-42

**Published:** 2008-04-23

**Authors:** Thomas Yau, Hilda Wong, Pierre Chan, MC To, Ronnie T Poon

**Affiliations:** 1 University Department of Medicine, Queen Mary Hospital, University of Hong Kong, Hong Kong; 2 University Department of Surgery, Queen Mary Hospital, University of Hong Kong, Hong Kong; 3 University Department of Pathology, Queen Mary Hospital, University of Hong Kong, Hong Kong

## Abstract

**Background:**

Hepatocellular carcinoma (HCC) is a common malignancy worldwide and treatment options are depended on the stage of the tumour. In general, the prognosis of HCC patients with extra-hepatic metastasis is poor. The most common sites of extra-hepatic metastasis are the lung, abdominal lymph nodes and bone.

**Case presentation:**

Here, we reported a 54-year-old man with an indolent clinical course of HCC. He had multiple extra-hepatic recurrences after initial hepatectomy for HCC and was benefited from repeated resections with prolonged survival. Eventually, he developed intramuscular recurrence in the thigh, which was initially mistaken as deep vein thrombosis.

**Conclusion:**

Selected patients with indolent disease course of HCC may benefit from repeated resections of extra-hepatic metastases with prolonged survival.

## Background

Hepatocellular carcinoma (HCC) is a common malignancy worldwide, especially in Southeast Asia where viral hepatitis is prevalent. Treatment options and thus prognosis are highly variable depending on the stage of the tumor. The prognosis of HCC patients with extra-hepatic metastasis is generally poor. The most common sites of extra-hepatic metastasis are the lung, abdominal lymph nodes and bone [1]. Rarely, HCC has been reported to metastasize to the breast [2], pituitary gland [3], gingiva and papillary muscle of the heart [4]. Intramuscular metastasis from HCC is indeed very rare. Here, we reported a 54-year-old patient with an indolent and interesting clinical course of HCC.

## Case presentation

**Figure 1:**
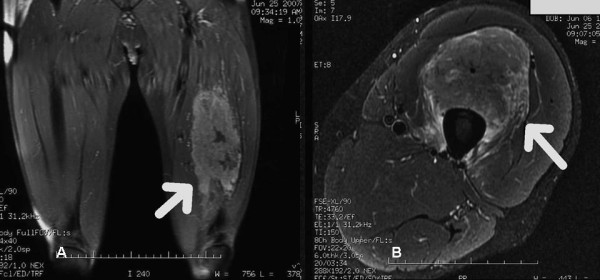
A and B: Magnetic Resonance Images of left thigh.

**Figure 2:**
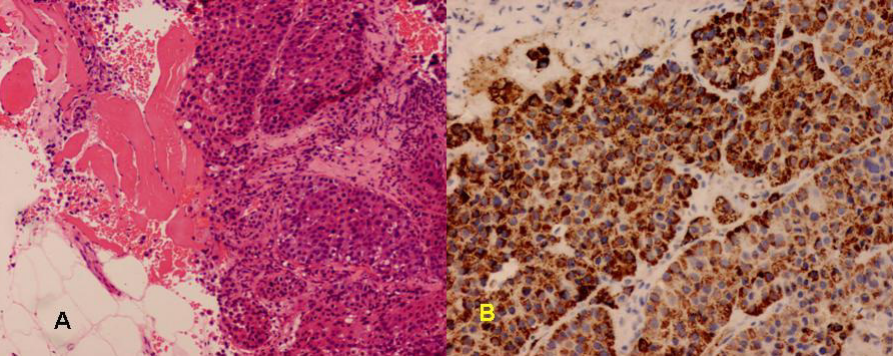
Histology of left thigh biopsy, A) Hematoxylin and eosin, B) Immunohistochemistry for HEPA.

A 54-year-man, who is a chronic hepatitis B carrier and with Child A cirrhosis, presented with weight loss in 2002. All along, he did not develop any variceal bleeding, ascites or hepatic encephalopathy. Blood tests showed elevated alpha-fetal protein (AFP) level of 458 ng/ml. Computer tomography (CT) imaging of the abdomen confirmed a 7.5 × 7 × 7.7 cm lesion in segment IVb of the liver. He underwent right tri-segmentectomy and at the time of surgery his Model for End Liver Disease (MELD) score was seven. Pathology showed a 8 × 5 × 4.5 cm HCC' with vascular invasion. He was followed up regularly with serum alpha-fetoprotein (AFP), chest x-ray and computed tomography (CT) scan every three months. Nine months after the initial operation, the disease recurred in the lung, with chest X-ray showing multiple lung shadows compatible with lung secondaries. Video-assisted thoracoscopic wedge resection of metastases in the right upper and lower lobes was performed. Pathology confirmed metastatic HCC in all resected lesions. However, six months after the operation, he developed brain metastasis with magnetic resonance imaging (MRI) of the brain showed a solitary metastasis in the left occipital region. Whole brain irradiation was offered, followed by craniotomy to resect the residual tumor in 2004. Later, left upper lobectomy was also performed for another intra-thoracic recurrence in the same year. After these operations, he remained clinically well with no clinical, serological and radiological evidence of recurrence till early 2007. He presented with progressive painful left thigh swelling for four months and was initially suspected to have deep vein thrombosis. Surprisingly, ultrasound study of the left thigh showed no evidence of such, but abnormally enlarged left rectus femoris with loss of normal muscle architecture. MRI suggested the soft tissue mass was likely to be intramuscular metastasis (Figure 1A–B). Ultrasound-guided biopsy revealed fibromuscular tissue infiltrated by tumor cells, which were immunohistochemically positive for HEPA (Fig. 2A–B). The overall picture was compatible with infiltration by metastatic HCC. Simultaneously, he was found to have a rise in AFP (177 ng/ml) and re-staging CT of the thorax and abdomen revealed multiple secondaries in all lobes of the lung. As a result, he was treated as widespread metastatic HCC by single agent sorafenib. After its commencement, the pain in left thigh had initially improved but soon resistant to treatment. He developed progressive disease with deteriorating condition and was finally succumbed of the disease in 2007.

## Discussion

To our knowledge, there are only very few reports in the literature regarding intramuscular HCC metastasis thus far [5,6]. In fact, despite the rich vascular supply of the muscle, it is rarely a site of metastasis in cancer patients [7]. This interesting phenomenon may be due to the contractility action of muscle, its local pH environment, and accumulation of lactic acid and other metabolites in the muscle [8].

The patient presented with a thigh metastasis. The other differential diagnosis in this case is deep venous thrombosis as HCC patients are found to have an increased risk for thromboembolism [9]. However, deep vein thrombosis is in fact a rare manifestation of hypercoagulable state in HCC patients.

In the present case, the patient has an indolent disease course of advanced HCC. His overall survival is largely enhanced by aggressive multiple resections of all the extra-hepatic sites of recurrence until finally the disease became widespread with involvement of the rare site of thigh muscle. In fact, in the literature, it is well recognized that treatment for extra-hepatic recurrence is important in prolonging survival especially in patients with well-controlled primary [10]. However, there is no convincing evidence to suggest that aggressive surgical resection of metastases from primary HCC is beneficial to all patients with metastatic HCC, albeit some studies suggested the benefits of such approach in highly selected HCC patients with distant metastases [11]. Nowadays, instead of aggressive metastatectomy, a novel multi-targeted kinase inhibitor – sorafenib offers new hope to this group of patients with unresectable metastases as it was shown to improve the overall survival with tolerable toxicities [12].

## Conclusion

Selected patients with indolent disease course of HCC may benefit from repeated resections of extra-hepatic metastases with prolonged survival. Eventually, the disease may develop recurrence in rare sites as illustrated in the present case and cause dilemma in the diagnosis and management.

## Competing interests

Dr Thomas Yau and Professor Ronnie Poon are the advisory board members for Bayer-Schering. There are no other conflicts of interest related to this manuscript.

## Authors' contributions

TY, HW, PC, and MCT, have made substantial contributions to conception and design, or acquisition of data, or analysis and interpretation of data: TY, HW, and RP have been involved in drafting the manuscript or revising it critically for important intellectual content. All authors have approved the final manuscript to be published
